# Olfactory receptor 10J5 responding to α-cedrene regulates hepatic steatosis via the cAMP–PKA pathway

**DOI:** 10.1038/s41598-017-10379-x

**Published:** 2017-08-25

**Authors:** Tao Tong, Sang Eun Ryu, Yeojin Min, Claire A. de March, Caroline Bushdid, Jérôme Golebiowski, Cheil Moon, Taesun Park

**Affiliations:** 1 Department of Food and Nutrition, Brain Korea 21 PLUS Project, Yonsei University, 50 Yonsei-ro, Seodaemun-gu Seoul, 120-749 South Korea; 2 Department of Brain and Cognitive Sciences, Daegu Gyeongbuk Institute of Science and Technology, Daegu, 711-873 South Korea; 3 Institut de Chimie de Nice, Université Nice Sophia Antipolis, Nice cedex 02, France; 4 Department of Molecular Genetics and Microbiology, Duke University Medical Center, Durham, North Carolina 27710 United States; 5 Convergence Research Advanced Centre for Olfaction, Daegu Gyeongbuk Institute of Science and Technology, Daegu, 711-873 South Korea

## Abstract

Ectopic expression and functions of odorant receptors (ORs) in the human body have aroused much interest in the past decade. Mouse olfactory receptor 23 (MOR23, olfr16) and its human orthologue, OR10J5, have been found to be functionally expressed in several non-olfactory systems. Here, using MOR23- and OR10J5-expressing Hana3A cells, we identified α-cedrene, a natural compound that protects against hepatic steatosis in mice fed the high-fat diet, as a novel agonist of these receptors. In human hepatocytes, an RNA interference-mediated knockdown of OR10J5 increased intracellular lipid accumulation, along with upregulation of lipogenic genes and downregulation of genes related to fatty acid oxidation. α-Cedrene stimulation resulted in a significant reduction in lipid contents of human hepatocytes and reprogramming of metabolic signatures, which are mediated by OR10J5, as demonstrated by receptor knockdown experiments using RNA interference. Taken together, our findings show a crucial role of OR10J5 in the regulation of lipid accumulation in human hepatocytes.

## Introduction

Olfactory receptors (ORs) are seven-transmembrane domain G protein-coupled receptors (GPCRs) that functions as chemosensors within the olfactory epithelium (OE) of the nose, where they detect the small molecules we perceive as odorants^1^. There are more than 1000 OR genes in mice and approximately 390 in humans, making them the largest receptor family in mammals^2, 3^. The odor information detected by this multiplicity of ORs is processed through a common signaling pathway: when an OR binds to its odorant, it activates a single G protein species, the olfactory trimeric G protein (G_olf_), which then activates the olfactory isoform of adenylate cyclase (Adcy3)^1, 4, 5^. Although these receptors were originally thought to be restricted to the nose^6^, it is now recognized that ORs are found in a variety of other tissues where they act as sensitive and selective chemoreceptors that influence many physiological processes^2–4, 7–10^. Pluznick *et al*. demonstrated that the olfr78/G_olf_/Adcy3 signaling system is present in the kidney, where it plays important functional roles in renin secretion and blood pressure regulation^7^. OR51E2 was reported to inhibit prostate cancer cell proliferation^8^, and OR2AT4 was reported to mediate human keratinocyte re-epithelialization during wound-healing process^9^. These observations lead to the intriguing consideration about the potential of targeting ORs in the treatment of human disease beyond their involvement in the fragrance industry.

Over the last decade, physiological functions of mouse olfactory receptor 23 (MOR23, olfr16) in nonsensory tissues or cells have been elucidated. Fukuda *et al*. demonstrated that MOR23 is functionally expressed in mouse spermatogenic cells and sperm, and its activation increases intracellular Ca^2+^ and regulates sperm motility^11^. MOR23 is also necessary for proper skeletal muscle regeneration because a loss of MOR23 leads to increased myofiber branching, commonly associated with muscular dystrophy^12^. Moreover, the human ortholog of MOR23 (OR10J5) has been demonstrated to be a key regulator of angiogenesis and to stimulate migration of human umbilical vein endothelial cells by activating the Ca^2+^-dependent AKT signal transduction pathway^13^. Unlike most of mammalian ORs, which lack an identified ligand, OR10J5 is known to recognize the synthetic odorant lyral^14, 15^. In the present study, α-cedrene, a sesquiterpene constituent of cedarwood oil derived from a number of *Cupressus* and *Juniperus* species of family *Cupressaceae*, was identified as an agonist of OR10J5. We demonstrate that α-cedrene along with lyral decreases triglyceride accumulation in human hepatocytes through the OR10J5–cAMP–PKA pathway.

## Results

### α-Cedrene alleviates hepatic steatosis in HFD-fed mice

**Figure 1:**
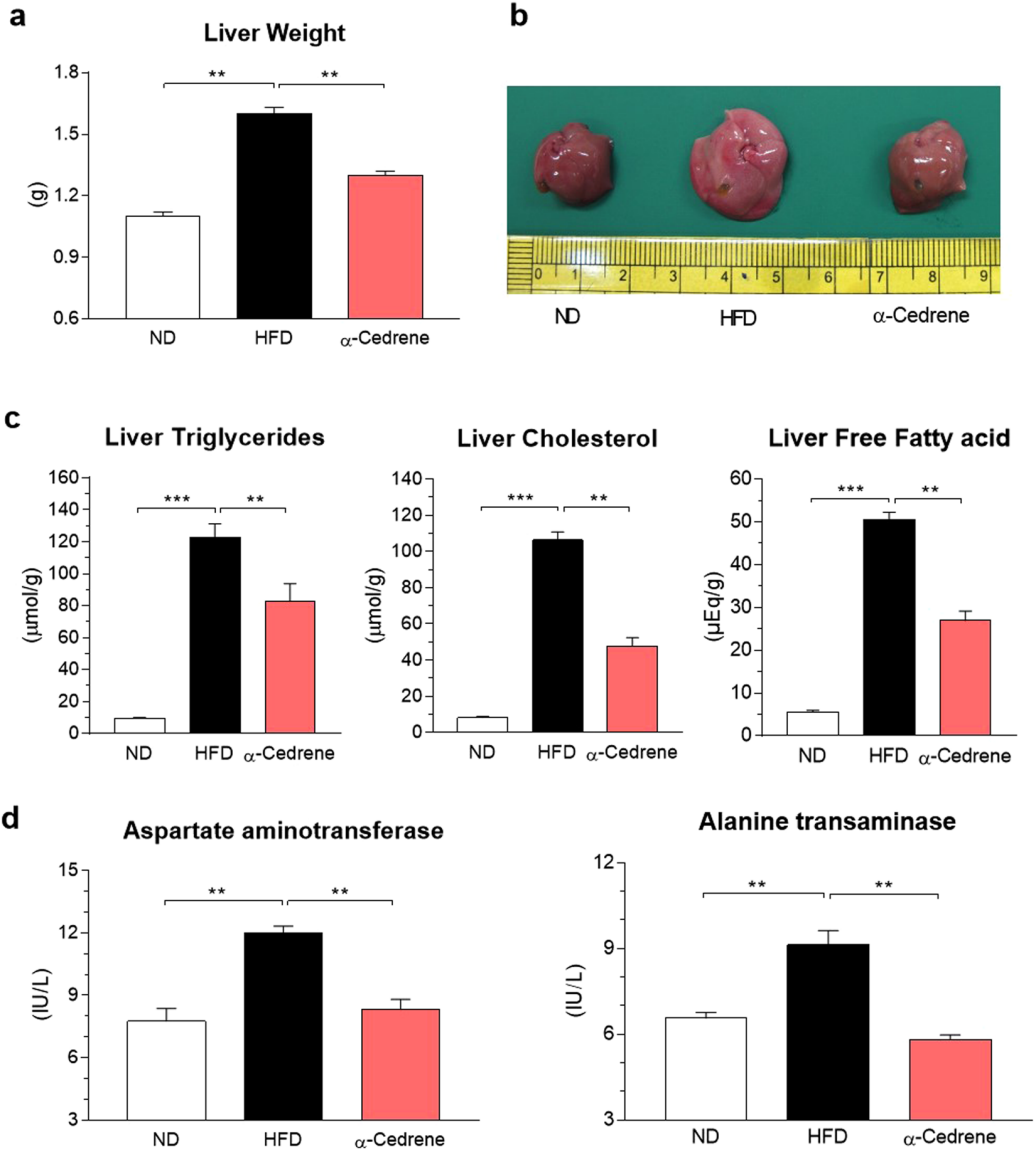
α-Cedrene alleviates hepatic steatosis in mice fed HFD. (**a**) The liver weights of mice fed the normal diet (ND), high-fat diet (HFD), or 0.2% (w/w) α-cedrene-supplemented diet. (**b**) Representative photographs of the liver from the ND-, HFD-, or α-cedrene-fed mice. (**c**) Hepatic content of triglycerides, cholesterol, and free fatty acids. (**d**) Plasma levels of aspartate aminotransferase and alanine transaminase. Values are presented as means ± SEM (n* = *8). Significant differences between groups are indicated by asterisks; *P < 0.05; **P < 0.01; ***P < 0.001.

α-Cedrene-fed mice showed significantly lower liver weights compared to HFD-fed mice (Fig. 1a). The livers of α-cedrene-fed mice maintained a healthy red color, whereas the livers of HFD-fed mice turned whitish (Fig. 1b). In line with these findings, biochemical analysis revealed that the hepatic accumulation of triglyceride, cholesterol, and free fatty acid induced by HFD was significantly decreased by α-cedrene treatment (Fig. 1c). Furthermore, the HFD-induced elevation of plasma aspartate aminotransferase and alanine transaminase, markers of liver injury, was significantly reversed by α-cedrene treatment (Fig. 1d).

### α-Cedrene induces cAMP accumulation and Ca^2+^ mobilization in olfactory sensory neuron

**Figure 2:**
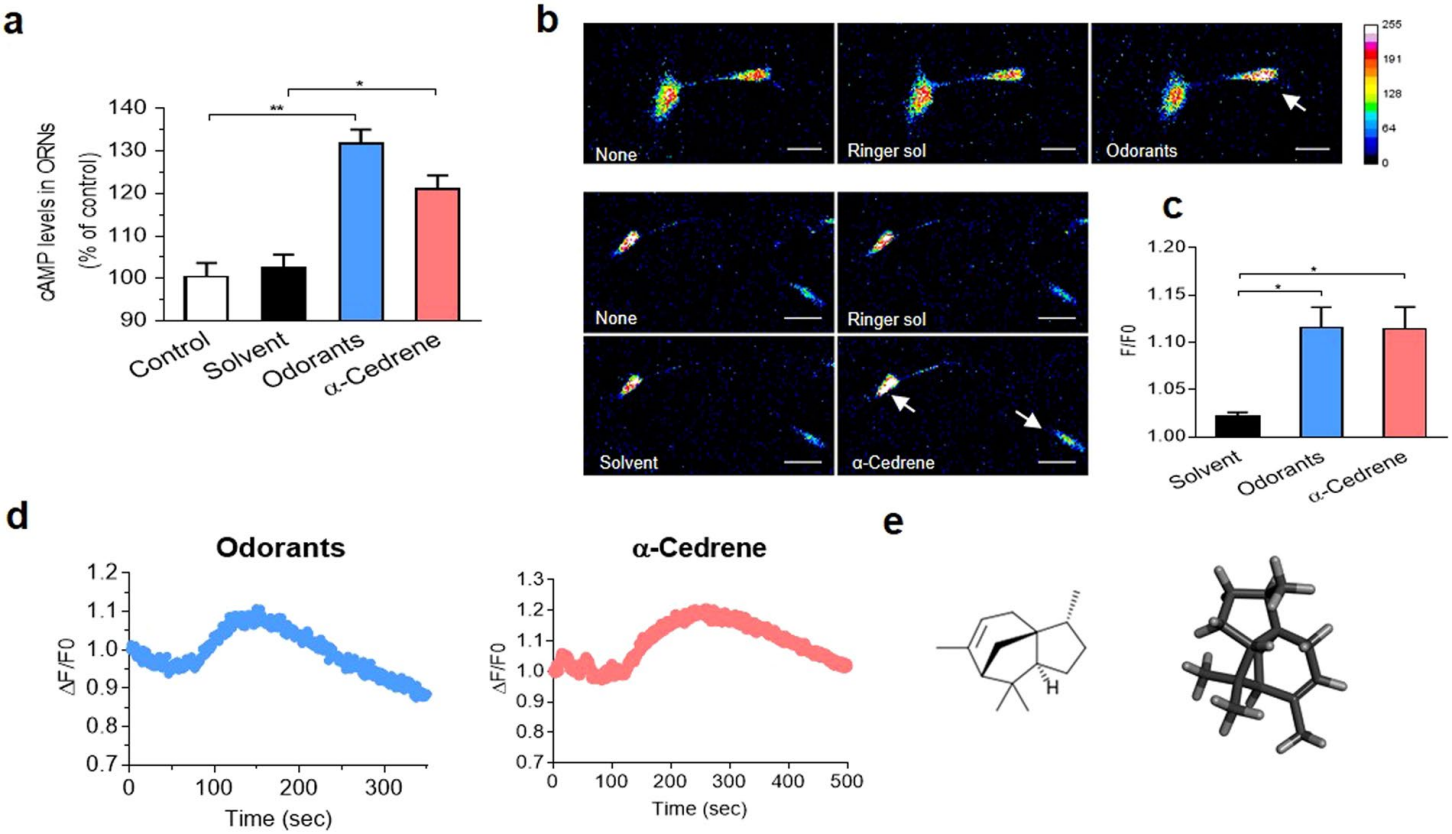
α-Cedrene induces cAMP accumulation and Ca^2+^ mobilization in olfactory sensory neuron. (**a**) Olfactory sensory neurons were pretreated with 3-isobutyl-1-methylxanthine (IBMX) for 30 min to inhibit phosphodiesterases, and intracellular cAMP levels were measured after 15 min of odorant mixture (10 μM) or α-cedrene (100 μM) stimulation. (**b**) Images of Fluo 3-AM fluorescent intensity (488 nm) from OSNs are presented in 16 colors to discriminate increased intracellular calcium levels. Every color refers to calcium concentration intensity and is indicated in a calibration bar. White arrows are pointed out to display the increases of intracellular calcium levels. All images were observed under the confocal microscope at 200x objectives (scale bar, 25 μm), and were analyzed using ImageJ software. (**c**) The elevated intracellular calcium concentrations (F; fluorescence intensity) of all responding cells were normalized with the control values (F0; fluorescence intensity of initiation) of Ringer solution treated cells. About 35% of total cells showed positive responses to the odorant mixture (n = 7 from 20 neurons), and 32% cells showed positive responses to the α-cedrene (n = 6 from 19 neurons). (**d**) The intracellular calcium levels were monitored every second until the recovery to the baseline level. Following treatment for 40 seconds with solvent, the OSNs were exposed to the odorant mixture (10 μM) or α-cedrene (100 μM). (**e**) Two-dimensional (2D) and 3D structures of α-cedrene obtained by means of the Gaussview software. Significant differences between groups are indicated by asterisks; *P < 0.05; **P < 0.01.

To determine whether α-cedrene activates ORs and relays olfactory signaling, we measured intracellular levels of cAMP, the major second messenger of olfactory signaling, and of Ca^2+^ in the primary rat olfactory sensory neurons (OSNs)^16, 17^. α-Cedrene increased intracellular cAMP level similarly to the action of odorant mixture in OSNs (Fig. 2a). Subsequently, treatment of OSNs with α-cedrene led to an increase in Ca^2+^ fluorescence intensity measured with a calcium indicator dye Fluo 3-AM in OSNs (Fig. 2b,c). We also monitored the temporal dynamics of Ca^2+^ in OSNs and observed α-cedrene caused a prolonged increase in intracellular Ca^2+^ levels that returned to the baseline level over a 500 second period (Fig. 2d). Taken together, these results indicated that α-cedrene elevated the intracellular cAMP and Ca^2+^ concentrations in manner similar to that of an odorant mixture (Fig. 2a–e), suggesting that α-cedrene is a possible ligand for ORs.

### α-Cedrene significantly increases the cAMP levels in MOR23- and OR10J5-expressing Hana3A cells

**Figure 3:**
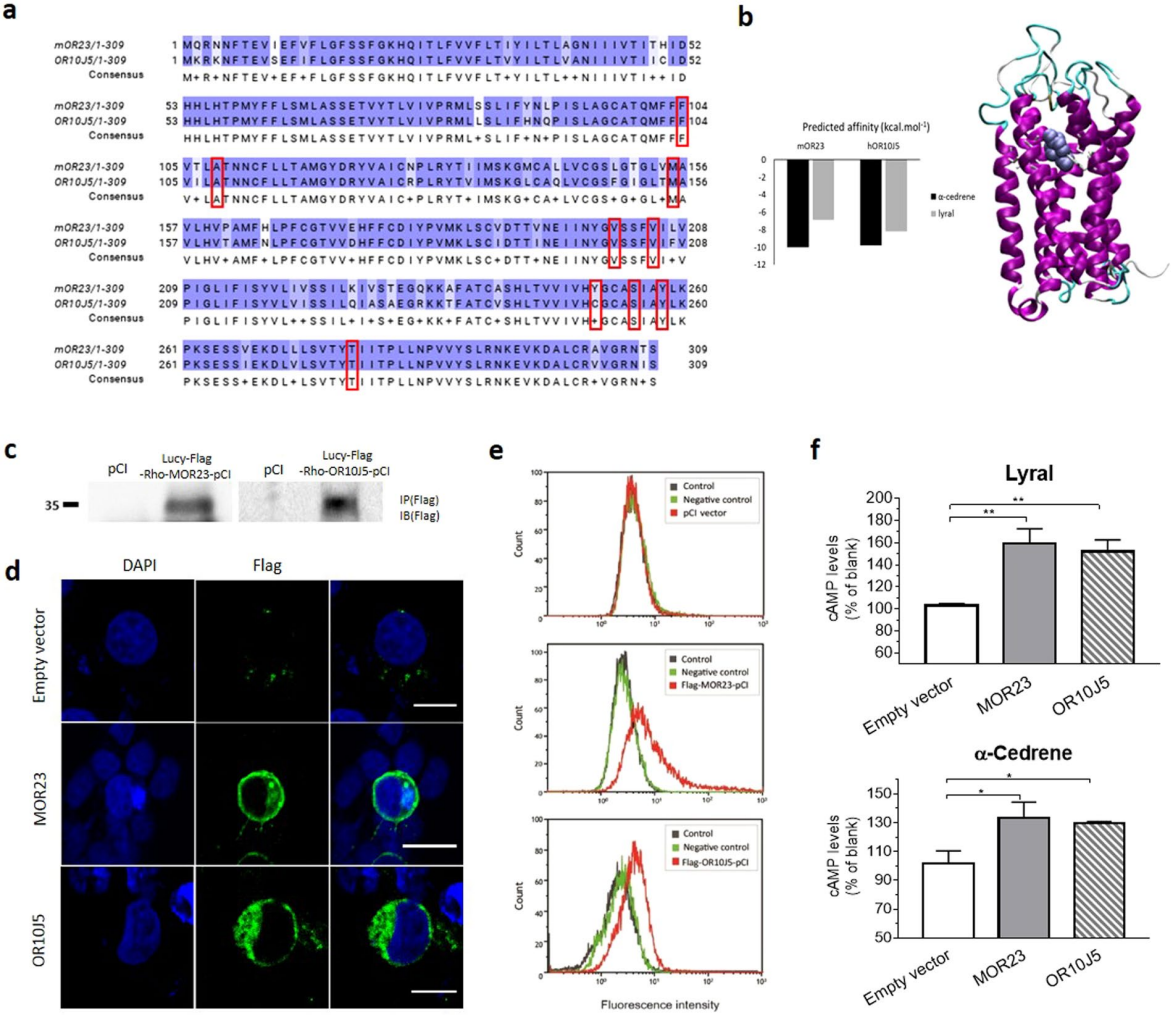
α-Cedrene significantly increases the cAMP levels in MOR23- and OR10J5-expressing Hana3A cells. (**a**) Sequence alignment of MOR23 and OR10J5. Identical residues are highlighted in blue, whereas different residues are not highlighted. Residues boxed in red represent amino acids which are predicted to point inside the binding cavity of both receptors. (**b**) α-Cedrene has better affinity than lyral for both MOR23 and OR10J5. Left panel: Docking scores for α-cedrene and lyral. The docking scores estimate free energy of association between the receptor and ligand. The more negative the score is, the stronger affinity the ligand has inside the cavity of the OR. Right panel: Helices are shown in purple and loops in blue and white. Residues lining the binding are shown in white, and the ligand is represented by violet balls (right). (**c**) Surface expressed FLAG-ORs were collected using FLAG-M2-magnetic beads. The released proteins were subjected to SDS-PAGE and immunoblotted with FLAG antibody. (**d**) Confocal imaging (×400) of transfected cells using the FLAG antibody to identify MOR23 or OR10J5 (green signal) revealed that these ORs are localized to the cell surface. Scale bars in empty vector and OR 10J5: 12 μm; scale bar in MOR23: 25 μm. (**e**) Flow cytometry analysis of cell-surface expression of tagged ORs in Hana3A cells. The fluorescence intensity among the green fluorescent protein-positive population was measured and plotted. (**f**) Intracellular cAMP levels were measured after 15 min of α-cedrene (100 μM) or lyral (100 μM) stimulation. Significant differences between groups are indicated by asterisks; *P < 0.05; **P < 0.01; ***P < 0.001.

To identify the putative ORs for α-cedrene, we screened the candidates from the mode-of-action by network identification (MNI) analysis by building their 3D structures and subjecting them to a docking protocol^18^. We tested whether α-cedrene could dock into each OR using the AutoDock Vina software^19^. We ranked the receptors by their affinity towards α-cedrene. MOR23, which is known to be functionally expressed in several non-olfactory systems^11–13^, was predicted as the top-ranked binder for α-cedrene (Fig. 3a,b). The sequence analysis of MOR23 and its human orthologue OR10J5 showed that they share 87% sequence homology and almost identical binding cavities with a large and highly hydrophobic character (Fig. 3a). This analysis suggested that they share the same chemical space of ligands (Fig. 3a).

We next compared the docking scores of α-cedrene and lyral, a synthetic odorant that binds to both MOR23 and OR10J5^14, 15^. Docking scores revealed that α-cedrene has better affinity than lyral for both ORs (Fig. 3b). On the basis of computational prediction, MOR23 and OR10J5 were predicted to be the best human OR candidates for driving the response of liver cells to α-cedrene stimulation. We next determined whether α-cedrene increases the cAMP levels in Hana3A cells heterologously expressing MOR23 or OR10J5 in a manner similar to that of lyral. We first prepared FLAG-tagged full-length constructs encoding MOR23 or OR10J5, and expressed them in Hana3A cells, and then assayed the ability of MOR23 and OR10J5 to reach the cell surface in the transfected cells by three different methods. Surface expression of MOR23 and OR10J5 was achieved as determined by western blotting analysis using FLAG antibody (Fig. 3c). Confocal imaging of transfected cells using the FLAG antibody to identify MOR23 or OR10J5 (green signal) confirmed membrane association of these expressed ORs (Fig. 3d). Additional flow cytometric analysis showed that cytometric profiles of MOR23 or OR10J5 are right-shifted, indicating ORs are expressed at the plasma membrane (Fig. 3e). Approximately 50% of all Hana3A cells showed surface expression of MOR23 or OR10J5 (Fig. 3c–e). Hana3A cells heterologously expressing MOR23 or OR10J5 showed a greater increase in lyral- or α-cedrene-stimulated cAMP production than those transfected with the empty vector (Fig. 3f).

### α-Cedrene reduces triglyceride concentration in HepG2 cells via the Adcy-cAMP pathway

**Figure 4:**
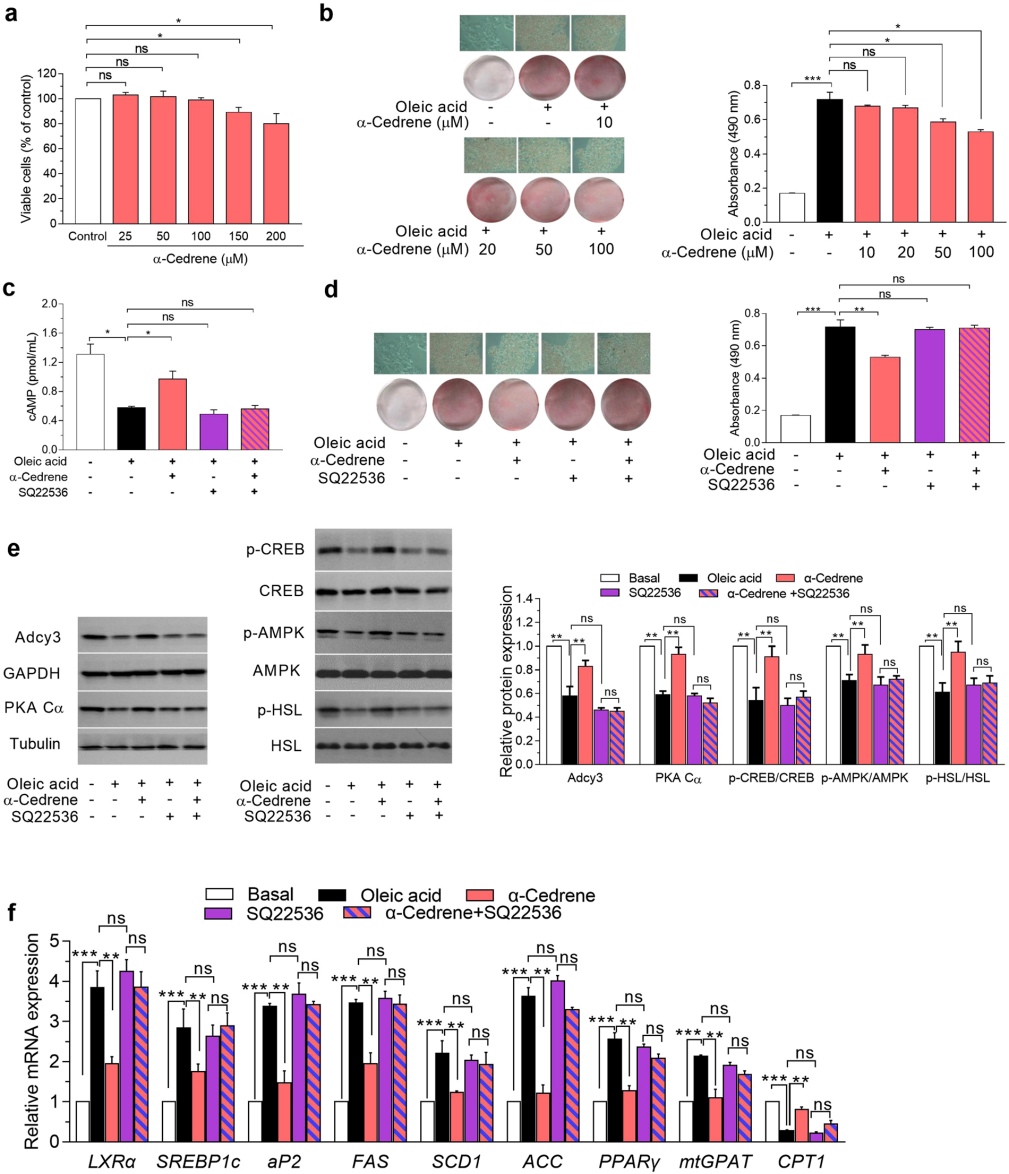
α-Cedrene reduces triglyceride concentration in HepG2 cells via the Adcy-cAMP pathway. (**a**) The percentage of viable cells was determined by an MTT assay. (**b**) Oil red O staining of HepG2 cells after stimulation by α-cedrene at the indicated concentration. Spectrophotometric quantification of Oil Red O-stained HepG2 cells is presented as means from three independent experiments for each group, and representative photomicrographs (×200) are shown in the left panel. (**c**) HepG2 cells were exposed to the Adcy inhibitor for 24 h with or without 100 μM α-cedrene, and levels of cAMP were measured. (**d**) Oil red O staining of HepG2 cells exposed to the Adcy inhibitor for 24 h with or without 100 μM α-cedrene. (**e**) Western blot analysis of Adcy3, PKA Cα, phosphor-CREB, phosphor-AMPK, and phosphor-HSL in HepG2 cells exposed to the Adcy inhibitor for 24 h with or without 100 μM α-cedrene. (**f**) Quantitative real-time PCR analysis of *LXRα*, *SREBP1c*, *aP2*, *SCD1*, ACC, *PPARγ*, *mtGPAT*, and *CPT-1* in HepG2 cells exposed to the Adcy inhibitor for 24 h with or without 100 μM α-cedrene. The full-length blots are presented in Supplementary Fig. 4. Significant differences between groups are indicated by asterisks; *P < 0.05; **P < 0.01; ***P < 0.001.

To obtain physiological proof that α-cedrene works in human the way that it does in a mouse model (Fig. 1), we tested the hypothesis that α-cedrene reduces the lipid accumulation in human hepatocytes. In 3-(4,5-dimethylthiazol-2-yl)-2,5-diphenyltetrazolium bromide (MTT) reduction assays with human hepatocytes (HepG2) exposed to α-cedrene, no inhibition of cell growth was observed after 24 h of treatment with 100 μM α-cedrene (Fig. 4a). To determine the effect of α-cedrene on hepatic lipid accumulation, we used a model of free fatty acid-induced cellular steatosis that recapitulates many key features of human hepatic steatosis. Increasing the concentration of α-cedrene (10–100 μM) led to a dose-dependent decline in intracellular lipid accumulation in HepG2 cells as determined by confocal microscopy after staining with Oil Red O (Fig. 4b). These findings were confirmed by colorimetric quantification of the intracellular triglyceride concentration in HepG2 cells exposed to various concentration of α-cedrene (Fig. 4b). Similarly, treatment of primary hepatocytes with α-cedrene (100 μM) also led to a significant decline in intracellular lipid accumulation (Supplementary Fig. 1). α-Cedrene also significantly increased intracellular cAMP levels in HepG2 cells (Fig. 4c). Next, we evaluated the effect of α-cedrene on the regulation of lipid accumulation in the presence of a commercially available inhibitor of adenylyl cyclases (Adcys) called SQ22536, because Adcy3 is indispensable for OR-mediated signaling pathways^20^. We found that the effects of α-cedrene on the regulation of intracellular levels of triglyceride and cAMP were completely blocked by treatment with SQ22536 (Fig. 4c,d). Treatment of human hepatocytes with α-cedrene (100 μM) also led to reprogramming of the metabolic signatures. The protein levels of Adcy3, PKA Cα, phosphor-CREB, phosphor-AMPK, and phosphor-HSL were found to be increased in α-cedrene-treated HepG2 cells (Fig. 4e). The mRNA levels of genes (*LXRα*, *SREBP-1c*, *PPARγ*, *FAS*, *SCD1*, *ACC*, and *mtGPAT*) involved in lipogenesis were decreased in α-cedrene-treated cells, with concomitant increase in mRNA expression of a gene related to fatty acid oxidation (Fig. 4f). These beneficial changes in protein and mRNA expression profiles elicited by α-cedrene were abrogated in the cells treated with the Adcy inhibitor (Fig. 4e,f). Furthermore, lyral induced physiological responses similar to those of α-cedrene in human hepatocytes (Supplementary Fig. 2a,b).

### A reduction in OR10J5 expression by specific siRNA abrogates the lipid-lowering effect of α-cedrene in HepG2 cells

**Figure 5:**
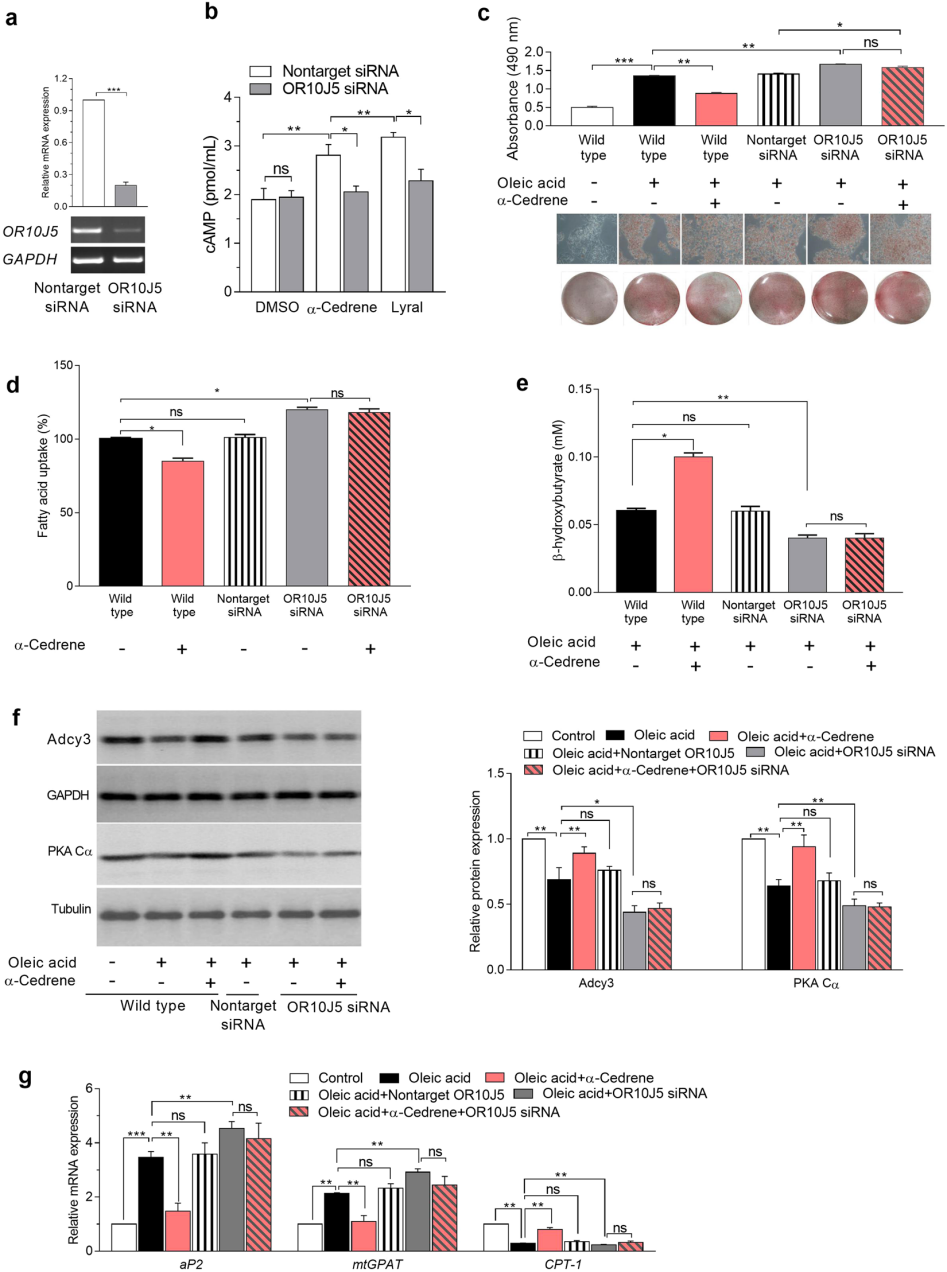
A reduction in OR10J5 expression by specific siRNA abrogates the lipid-lowering effect of α-cedrene in HepG2 cells. (**a**) OR10J5 was knocked down in HepG2 cells by siRNA transfection. RT-PCR indicates that OR10J5 expression was decreased by anti-OR10J5 siRNA. (**b**) HepG2 cells from control or siRNA cultures were treated with α-cedrene (100 μM) or lyral (100 μM) for 24 h, and levels of cAMP were measured. (**c**) Oil red O staining of HepG2 cells transfected with nontargeting (control) siRNA or anti-OR10J5 siRNA for 24 h with or without 100 μM α-cedrene. (**d**) Fatty acid uptake by HepG2 transfected with control siRNA or anti-OR10J5 siRNA for 24 h with or without 100 μM α-cedrene. (**e**) The concentrations of β-hydroxybutyrate in the medium of HepG2 cells transfected with control siRNA or anti-OR10J5 siRNA for 24 h with or without 100 μM α-cedrene. (**f**) Western blot analysis of Adcy3 and PKA Cα in HepG2 cells transfected with nontargeting siRNA or anti-OR10J5 siRNA for 24 h with or without 100 μM α-cedrene. (**g**) Quantitative real-time analysis of *aP2*, *mtGPAT*, and *CPT-1* in HepG2 cells transfected with nontargeting siRNA or anti-OR10J5 siRNA for 24 h with or without 100 μM α-cedrene. The full-length gels/blots are presented in Supplementary Figs 5 and 6. Significant differences between groups are indicated by asterisks; *P < 0.05; **P < 0.01; ***P < 0.001.

To validate the possibility that the effectiveness of α-cedrene in reducing lipid accumulation in hepatocytes derives from activation of OR10J5-mediated signaling pathways, we knocked down OR10J5 in HepG2 cells using OR10J5-specific small interfering RNA (siRNA; a pool of four OR10J5-specific oligonucleotides) (Fig. 5a,b). The OR10J5 depletion significantly increased intracellular lipid accumulation in HepG2 cells (Fig. 5c), along with upregulation of lipogenic and downregulation of genes related to fatty acid oxidation (Fig. 5f,g). Following α-cedrene stimulation, the HepG2 cells showed a significant decrease in fatty acid uptake (Fig. 5d) and increase in ketone body release (Fig. 5e). The beneficial changes in both lipid metabolism and gene expression profiles elicited by α-cedrene in oleic acid-treated HepG2 cells were abrogated by the siRNA-mediated knockdown of OR10J5 (Fig. 5c–g), indicating that α-cedrene specifically exerted its effect on the regulation of lipid accumulation via OR10J5 activation. In the meantime, α-cedrene treatment had no effect on gene expression of OR10J5 in HepG2 cells (Supplementary Fig. 2c).

## Discussion

Using MNI analysis, which is a validated means for the identification of targets and associated pathways of compounds^21–24^, we have previously demonstrated that ORs might be the potential genetic mediators of HFD-induced obesity progression in adipose tissues and muscles^25^. Here, we used a phenotypic approach (i.e., exposing HFD-fed mice to drug candidates) to look for small molecules that could attenuate hepatic steatosis. Through this process, we found that administration of α-cedrene led to a robust reduction in the hepatic lipid content in HFD-fed mice (Fig. 1). Thus, we reasoned that the protective effect of α-cedrene against hepatic steatosis could be mediated by the activation of ORs expressed in the liver. To identify the putative molecular targets for α-cedrene, we studied the molecular changes in the liver in response to α-cedrene by MNI analysis. The results led to the identification of several ORs as promising candidates. Subsequently, we used *in silico* docking to predict the binding conformation and affinity of α-cedrene to these ORs at the atomic level. This analysis yielded MOR23 as the top-ranked binder for α-cedrene. These results support the idea that α-cedrene may exert its protective effect against hepatic steatosis through an MOR23-mediated pathway.

The total amount of lipids in hepatocytes mainly depends on fatty acid synthesis and oxidation^26^. The cAMP/PKA signaling pathway plays a significant role in regulating hepatic lipid homeostasis. In most cases, cAMP is produced following GPCR-mediated activation of the stimulatory Gα protein (Gα_s_), which in turn activates Adcy that catalyzes the conversion of ATP to cAMP^27^. The accumulation of cAMP activates cAMP-dependent protein kinase (PKA), which phosphorylates several proteins, such as adenosine monophosphate-activated protein kinase (AMPK)^28^, cAMP-response element binding protein (CREB)^29^, and hormone-sensitive lipase (HSL)^30^. Phosphorylated AMPK then inhibits liver X receptor α (LXRα), resulting in the downregulation of sterol regulatory element-binding protein 1c (SREBP-1c) and its target genes including acetyl CoA carboxylase (ACC), fatty acid synthase (FAS), and stearoyl CoA desaturase-1 (SCD-1)^31–33^. Moreover, phosphor-CREB inhibits the expression of nuclear hormone receptor PPARγ, a key regulator of lipogenic genes^34^. In parallel, upon cAMP/PKA-induced phosphorylation, HSL translocates to the surface of lipid droplets to commence fatty acid oxidation program^30^.

In response to the stimulation of adenylyl cyclase through a G-protein coupled receptor, the second messenger, cAMP, is generated. Binding of cAMP to the regulatory subunit of PKA causes the catalytic subunit to be unleashed so that it can phosphorylate its protein substrates^35^. In the present study, treatment of HepG2 cells with α-cedrene increased the protein levels of Adcy3 and catalytic subunit of PKA (PKA Cα). By contrast, these α-cedrene-induced changes were markedly blocked by the OR10J5 knockdown in the HepG2 (Fig. 5f), indicating that α-cedrene affects the protein expression of Adcy3 and PKA Cα in an OR10J5-dependent manner. This finding is in line with the recent reports indicating that protein levels of Adcy3^36, 37^ and PKA Cα^38^ was upregulated in response to agonist-induced stimulation of a GPCR in mouse liver tissue and bone marrow stromal cells, respectively. Nevertheless, it remains to be determined how α-cedrene that acts through OR10J5 impacts Adcy3 and PKA Cα expression.

In the present study, the depletion of OR10J5 or pharmacological inhibition of Adcy3, which is downstream signal transducing component of the olfactory signaling machinery, significantly increased intracellular lipid accumulation in HepG2 cells, along with upregulation of lipogenic genes and downregulation of genes related to fatty acid oxidation. This finding is consistent with the results of our previous report, which showed that *Adcy3* heterozygous null mice have significantly higher hepatic triglyceride, cholesterol, and fatty acid levels than WT mice^39^.

ORs comprise nearly 50% of the ~800 GPCRs in humans, yet ~90% of human ORs remain orphan receptors with unknown ligands^40^. Previous analysis of a limited number of ORs that have been functionally matched with their cognate ligands revealed that ORs bind odorants combinatorially: each OR can respond to multiple odorant molecules, albeit with varying response amplitudes, and a given odorant can elicit responses from multiple OR types^41^. Moreover, recent studies suggested that a single OR can not only recognize structurally related odorants but also is activated by a variety of odorants with diverse chemical structures^42^. For example, mOR-EG recognizes approximately 25 structurally related agonists with different potencies^43–46^, whereas MOR174-4 responds to a few structurally different odorants^42^. It is therefore not surprising that, in the present study, OR10J5 responds to the two structurally different odorants: α-cedrene and lyral.

A given cell type in non-olfactory tissues tend to express more than one OR gene, and a specific OR is known to be functionally expressed in a wide range of tissues and cell types where it performs diverse functions. For instance, MOR18-2 (olfr78) is expressed in renal juxtaglomerular apparatus, where it participates in the regulation of renin secretion and blood pressure regulation^7^. Furthermore, a recent study showed that MOR18-2 is highly and selectively expressed in oxygen-sensitive glomus cells of the carotid body and acts as a hypoxia sensor in the breathing circuit by sensing lactate produced when oxygen levels decline^10^. Therefore, given the presence of OR10J5 in diverse tissues reported previously^13^, it is conceivable that OR10J5 would reveal additional unexpected functions in various peripheral tissues. In addition to attenuating hepatic steatosis, administration of α-cedrene significantly reduced the body weight gain of HFD-fed mice without affecting the food intake (Supplementary Fig. 3a–c). Future studies will be necessary to determine the specific effects of OR10J5 in different cell types involved in metabolic disorders, such as adipocytes and myocytes.

**Figure 6:**
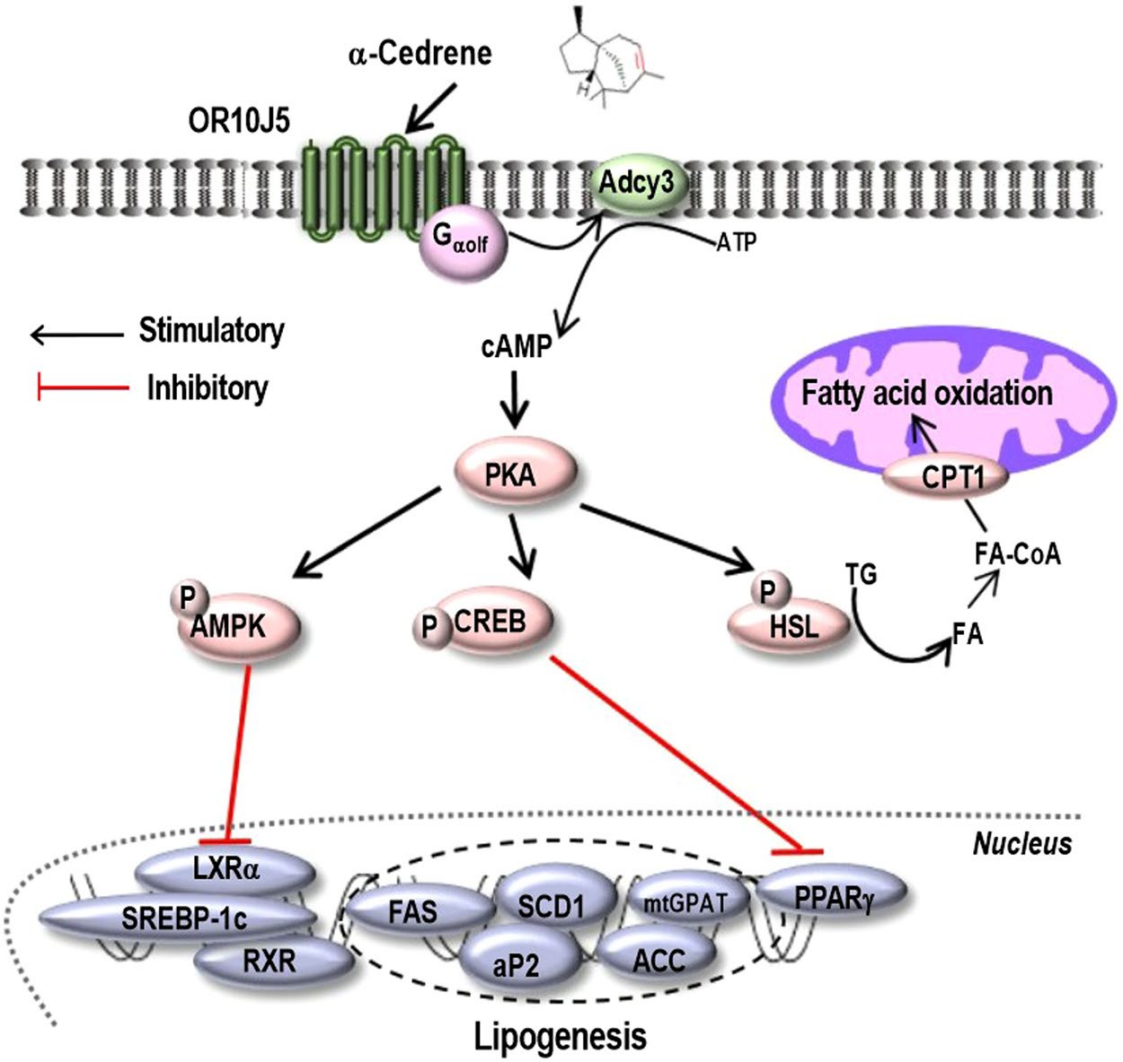
A schematic diagram illustrating the proposed mechanism by which α-cedrene regulates lipogenesis and fatty acid oxidation in hepatocytes.

In summary, we found that α-cedrene significantly increases the cAMP levels in MOR23- and OR10J5-expressing Hana3A cells and that treatment of human hepatocytes with α-cedrene results in significant reduction (by 36%) of lipid contents, which was abrogated in OR10J5 deprived cells. In the present study, lyral, a known synthetic ligand for MOR23 and OR10J5, induced physiological responses similar to those of α-cedrene in human hepatocytes. In line with these direct physiological responses, treatment of human hepatocytes with α-cedrene led to reprogramming of metabolic signatures: the levels of Adcy3 and of genes involved in fatty acid oxidation were increased in α-cedrene-treated cells, with concomitant downregulation of lipogenic genes. The beneficial changes in gene expression profiles elicited by α-cedrene were abrogated by the OR10J5 knockdown in the cells. Taken together, our findings point to a novel and crucial role of OR10J5 in the regulation of lipid accumulation in human hepatocytes as illustrated in Fig. 6.

## Materials and Methods

### Reagents

The antibody against FLAG tag was purchased from Sigma-Aldrich (F1804, St. Louis, MO, USA; 1:1,000 dilution). The antibodies against the following proteins were purchased from Cell Signaling Technology (Danvers, MA, USA): PKA Cα (#4782; 1:1,000 dilution), phosphor-CREB (#9198; 1:1,000 dilution), CREB (#9197; 1:1,000 dilution), phosphor-AMPK (#2531; 1:1,000 dilution), AMPK (#2532; 1:1,000 dilution), phosphor-HSL (#4139; 1:500 dilution), HSL (#4107; 1:500 dilution), and GAPDH (#2118; 1:5,000 dilution). The antibody against Adcy3 (sc-588; 1:200 dilution) was purchased from Santa Cruz Biotechnology (Dallas, TX, USA). A horseradish peroxidase (HRP)-conjugated anti-rabbit IgG antibody (1:5,000 dilution; Santa Cruz Biotechnology cat. # sc-2004; secondary antibody) was used for immunoblotting procedures, and an Alexa 488-conjugated anti-mouse antibody (Abcam, Cambridge, UK secondary antibody) was used for live-cell detection. Oleic acid, fatty acid free-bovine serum albumin (BSA), the MTT solution, Oil red O, and lyral were purchased from Sigma-Aldrich. α-Cedrene (batch No. KDCB212DA01, purity: 99.6%) was supplied by Kwang Dong Pharmaceutical Co. (Seoul, South Korea). An odorant mixture (2-isobutyl-3-methoxypyrazine, citralva, and isovaleric acid) was dissolved in the medium directly from 1 M stock.

### Animals and diets

Male C57BL/6 N mice (5 weeks old) were purchased from Orient Bio (Gyeonggi-do, South Korea) and were housed in standard cages in a room at 21 ± 2.0 °C, 50 ± 5% relative humidity, and a 12-h light/12-h dark cycle. All the mice consumed a commercial diet and tap water ad libitum for 1 week prior to their distribution into three weight-matched groups (n = 8 per group): the normal diet (ND), high-fat diet (HFD), and 0.2% (w/w) α-cedrene-supplemented diet. ND was a purified diet based on the AIN-76 rodent feed. HFD was identical to ND, except that 200 (g fat)/kg (170 g lard plus 30 g corn oil) and 1% cholesterol were added to it. The α-cedrene-supplemented diet was identical to HFD but contained 0.2% (w/w) α-cedrene. The experimental diets were provided *ad libitum* for 10 weeks in the form of pellets.

The mice were weighed every 7 days, and their food intake was recorded daily during the feeding period. At the end of the experimental period, the animals were anesthetized with ether after 12-h fasting. Blood was drawn from the abdominal aorta into an EDTA-coated tube, and plasma was separated by centrifuging the blood at 2000*g* for 15 min at 4 °C. The entire liver was dissected out, weighed, and snap-frozen in liquid nitrogen prior to storage at −70 °C. All animal experiments were performed in accordance with the Korea Food and Drug Administration guidelines. All experimental protocols were reviewed and approved by the Institutional Animal Care and Use Committee of the Yonsei Laboratory Animal Research Center.

### Biochemical analysis

Hepatic lipids were extracted by the method of Folch *et al*.^47^. Briefly, 100-mg aliquots of tissue were homogenized with 1 mL distilled water for 5 min. A fresh 2.5 mL solvent mixture containing chloroform and methanol (2:1, v/v) was added to the homogenate and centrifuged at 3000 g for 10 min to separate the phases. The lower phase was removed carefully and transferred to a new tube. Additional solvent was added to the upper phase and the pellet in a final volume of 6.25 mL was vortexed, then centrifuged at 3000 g for 10 min. The lower phase was repeatedly collected with 1.5 mL of washing buffer containing chloroform, methanol and 0.05% CaCl_2_ (3:48:47, v/v/v). After vortexing and centrifugation, the lower phase containing lipids was evaporated under a nitrogen stream and the dried lipid residues were then dissolved in 2 mL of ethanol. The concentrations of cholesterol, triglycerides, and free fatty acids in the hepatic-lipid extracts were measured using enzymatic kits (Asan Pharmaceutical Co., Seoul, Korea**)** according to the manufacturer’s protocols. Alanine transaminase and aspartate aminotransferase levels were determined on an automatic analyzer (Express Plus, Chiron Diagnostics, East Walpole, MA, USA).

### cAMP assay

OSNs were plated at a density of 2 × ${10}^{6}$ cells/mL in laminin-coated plates, and OR-expressing Hana3A cells were plated at a density of ${10}^{6}$/mL in poly-D-lysine–coated plates 24 h prior to the assay. The intracellular cAMP levels were measured by means of the enzyme immunoassay Parameter® kit (R&D Systems, Minneapolis, MN, USA). The principle of the cAMP assay is competitive binding. The cAMP within the sample competes with a fixed amount of HRP-labeled cAMP for sites on the monoclonal antibody. cAMP was quantified at 450 nm.

In case of HepG2 cells, they were lysed with 0.1 M HCl at 500 μl/well. The lysates were immediately processed with the Direct cAMP ELISA kit (Enzo Life Sciences, Farmingdale, NY, USA) to measure the intracellular concentration of cAMP. The data were analyzed on a microplate reader (Versa Max, Molecular devices, CA, USA) at 405 nm.

### DNA constructs and cloning

The full-length genomic DNA of the OR gene was obtained from mouse tissue and HEK293 cells by means of the following specific primers: 5′-TCACGCGTATGCAGAGAAATAACTTCAC-3′ and 5′-AAGCGGCCGCTTAAGAAGTGTTTCTGCCCA-3′ for MOR23, and 5′-TCACGCGTATGAAGAGAAAGAACTTCAC-3′ and 5′-AAGCGGCCGCTTAAGAAATATTTCTGCCCACAACT-3′ for OR10J5. Each OR sequence was subcloned between Mlu I and Not I restriction enzyme sites of the pCI mammalian expression vector. The pCI vector containing N-terminal Rho tags and RTP1S-pCI constructs was a kind gift from Dr. Hiroaki Matsunami (Duke University, USA). To promote surface expression of ORs, a leucine-rich 17-amino acid cleavable signal peptide (MRPQILLLLALLTLGLA) was followed by the FlAG and Rho sequences at the N-terminus of ORs, according with the method of Shepard *et al*.^48^. All constructs were subjected to DNA sequencing to confirm its identity.

### Cell culture

OSNs were obtained from Sprague-Dawley rats as previously described with some modifications^49^. The cells were plated in tissue culture dishes coated with 25 μg/mL laminin (BD Bioscience. San Diego, CA, USA) in modified Eagle’s medium containing D-valine (MDV, Welgen Inc., Worcester, MA, USA). The cultures were placed in a humidified 37 °C incubator at 5% CO_2_. On the second day of cultivation and every day thereafter, the cells were grown in MDV containing 15% of dialyzed fetal bovine serum (Gibco, Rockville, MD, USA), gentamicin, kanamycin and 2.5 ng/mL nerve growth factor. Two days prior to the use of the cells in experiments, the culture medium was changed to the medium without nerve growth factor.

HepG2 human hepatoma cells were purchased from ATCC (Manassas, VA, USA) and cultured in modified Eagles’s medium (MEM; Hyclone, Logan, UT, USA) plus 10% of fetal bovine serum (FBS; Gibco-BRL, Grand island, NY, USA), penicillin/streptomycin, and L-glutamine at 37 °C and 5% CO_2_.

Cryopreserved plateable mouse hepatocytes were purchased from CellzDirect^TM^ (Thermo Fisher Scientific, Waltham, MA, USA). Upon arrival, the cells were plated according to the manufacturer’s instructions. That is, cells in storage medium were added to 48 mL of warmed thawing medium and centrifuged at 55 g for 3 min at room temperature. The resulting pellet was re-suspended in 4 mL of plating medium. Cells were then seeded in a 6-well collagen coated plate and incubated with the plating medium at 37 °C with 5% CO_2_ for 6 h to allow the cells to adhere. The plating medium was then replaced with incubation medium and further incubated for 16 h prior to experiments.

### Calcium imaging

OSNs were cultured in laminin-coated 12-well mm dishes at a density of ${10}^{6}$/mL for 3 days, and labeled with 3 mM Fluo-3 AM reagent (F-23915; Molecular Probes, Eugene, OR, USA) at 37 °C for 30 min. After that, the medium was replaced with Ringer’s solution, and the cells were visualized under a Zeiss LSM 7 Live microscope (Carl Zeiss, Jena, Germany).

### Model building and analysis of binding cavity

To characterize the features of the binding cavity, the Modeller^50^ software was used to build models for MOR23 and for OR10J5, following a published method^18^. Sequences of these receptors were aligned with all 396 sequences of human ORs and nine sequences of crystallized GPCRs (templates). The templates that was used are all X-ray elucidated structures of class A GPCRs including bovine rhodopsin receptor (PDB ID: 1U19), human β2 adrenergic receptor (PDB ID: 2RH1), turkey β1 adrenergic receptor (PDB ID: 2VT4), human chemokine receptors CXCR4 (PDB ID: 3ODU) and CXCR1 (PDB ID: 2LNL), human dopamine receptor D3 (PDB ID: 3PBL), human adenosine A2A receptor (PDB ID: 2YDV), human histamine H1 receptor (PDB ID: 3RZE), and muscarinic acetylcholine receptor M2 (PDB ID: 3UON).

Highly conserved motifs in ORs were regarded as contraints for the alignment: GN in helix 1, PMYFFLxxLSxxD in helix 2, MAYDRYVAIC in helix 3, SYxxI in helix 5, KAFSTCxSH in helix 6, LNPxIY in helix 7 and a pair of conserved cysteines 97–178, which constitutes a known disulfide bridge between the beginning of helix 3 and the extracellular loop 2. Four crystallized structures (1U19, 3ODU, 2YDV and 2LNL) were selected as templates to build mOR23 and OR10J5 by homology modeling in the Modeller software. Five models were obtained, and the one fulfilling several constraints (binding cavity sufficiently large, all TMs folded as α-helices, and a small helical structure in Cter) was kept.

### Affinity prediction via docking

We docked the structure of α-cedrene and of lyral inside each of the ORs using the AutoDock Vina software^19^. The molecular docking approach can be used to model the interaction between a small molecule and protein at the atomic level, which allowed us to predict the position and orientation of a ligand in the binding cavity of target proteins. The docking scores that were obtained allowed us to estimate free energy of the association between the receptor and ligand. In a docking simulation, we used semi-flexible docking protocols in which the defined binding-site residues inside the cavity were treated flexible whereas the others were kept rigid. The predicted affinity is given in kcal·mol^−1^.

### Surface expression of ORs

Hana3A cells were plated at a density of 3.5 × ${10}^{5}$/mL to the poly-D-lysine coated plate 24 h prior to the transfection. MOR23 and OR10J5 (LucyFlagRho-OR-pCI) were transfected into Hana3A cells together with pCI-RTP1s by means of Lipofeactamine 2000 reagent (Invitrogen, Carlsbad, CA, USA). After 24 h of transfection, the cells were detached from the plate and split into the poly-D-lysine-coated 12-mm dishes at a density of ${10}^{5}$/mL for immunocytochemistry analysis. For flow cytometry, the cells were surface stained and washed with phosphate-buffered saline (PBS) containing 2% of FBS and 15 mM NaN3 at 4 °C. For both purposes, the cells were incubated with a mouse monoclonal antibody against the FLAG tag. The cells were fixed with 4% paraformaldehyde prior to the staining with the primary antibody for immunocytochemistry. In addition, the cells were incubated with an Alexa 488-conjugated anti-mouse IgG antibody (Abcam, Cambridge, UK; secondary antibody). The surface-expressed ORs were examined under a Zeiss LSM 700 confocal microscope (Carl Zeiss) and quantified on a Gallios Flow Cytometer (Beckman Coulter, Brea, CA, USA). Kaluza software was used for the analysis.

### The MTT assay

HepG2 cells were seeded in a 96-well plate at a density of 2 × 10^4^/mL and incubated overnight. The cells were then incubated with different concentrations of α-cedrene (0, 25, 50, 100, 150, or 200 μM) for 24 h. At the end of the incubation, 50 μl of the MTT solution (5 mg/mL, Sigma-Aldrich) was added to each well, and the plates were incubated for 4 h at 37 °C. Then, 50 μL of DMSO was added to each well and incubated for 30 mins at room temperature. Cell viability was evaluated by measuring the mitochondria-dependent conversion of the yellow tetrazolium salt MTT to purple formazan crystals by metabolically active cells. The optical density was assessed on a Microplate Reader (Versa Max) at 570 nm.

### Lipid accumulation and Oil Red O staining

HepG2 cells were cultured in 6-well plates. The medium was changed in serum-free MEM, followed by incubation of the cells with a 0.5 mM oleic acid (OA) solution for 24 h. Plates were washed twice with PBS and fixed with 10% buffered formalin for 1 h at room temperature. The cells were then stained with a filtered Oil Red O solution (0.5% Oil Red O in isopropyl alcohol) for 30 min in a 37 °C incubator and were washed twice with distilled water. After washing and complete drying, 500 μl of isopropanol was added into each well, followed by incubation at room temperature for 10 min to release Oil Red O from steatosis staining. The extraction solution was transferred to another 96-well plate which was subjected to measurement of optical density at a wavelength of 490 nm using a microplate reader (Versa Max).

### Fatty acid uptake and β-hydroxybutyrate measurement

Fatty acid uptake was measured using a fluorescent free fatty acid uptake assay kit (ab176768, Abcam, Cambridge, UK), following the manufacturer’s instructions. Briefly, cells were seeded at a density of 50,000 cells per well in a 96-well plate, and the following day, cells were deprived of serum for 1 h. Cells were then incubated with labeled C12 fatty acid at room temperature, and fluorescence per well (excitation: 485 nm, emission: 515 nm) was measured using a fluorescence plate reader (Victor X5, Perkin Elmer Life Sciences, Ontario, Canada). For β-hydroxybutyrate measurement, cells were subjected to α-cedrene (100 μM) and oleic acid (0.5 mM) treatment for 24 h and consequent β-hydroxybutyrate release in the medium was measured using the ketone body assay kit (MAK134, Sigma-Aldrich).

### RNA extraction and PCR

Total RNA was extracted form HepG2 cells using the TRIzol reagent (Life Technology, Carlsbad, CA, USA) according to the manufacturer’s instructions. cDNA synthesis was performed with 1 μg of total RNA, 5× RT buffer, 2.5 mM each dNTP, 0.1 M dithiothreitol, 200 U/μL reverse transcriptase, and 40 U/μL RNase inhibitor at 37 °C for 2 h. For semi-quantitative PCR, the amounts of mRNA were measured using the 2× PCR Master Mix (Intron, Seoul, Korea) with *GAPDH* as an internal control. Quantitative PCR was performed (after reverse transcription of RNA) using iQ SYBR green supermix (Bio-Rad, Hercules, CA, USA) with the CFX Connect™ Real-Time PCR Detection System (Bio-Rad). All gene expression data were normalized to GAPDH. Primer sequences are listed in Supplementary Table 1. The results on the optical density ratio of a target gene to *GAPDH* are presented as mean ± SEM of at least three different experiments.

### Protein extraction and western blotting

OR-transfected cells were lysed with lysis buffer containing protease and phosphatase inhibitors (0.5% TRITON X-100, 100 mM NaCl, 50 mM Tris-HCl, 5 mM EDTA). Next, 1 mg of a lysate was gently agitated with anti-FLAG® M2 magnetic beads (Sigma-Aldrich) for 2 h at room temperature. Immunoprecipitated FLAG-tagged proteins were detected by means of an anti-FLAG mouse monoclonal antibody.

HepG2 cells were harvested in lysis buffer and incubated at −20 °C for 20 min. The lysates were cleared by centrifugation at 4 °C for 10 min at 12,100 g, and protein concentrations were determined using the Bradford dye (Bio-Rad, Hercules, CA, USA). The obtained protein samples were separated by 10% SDS-PAGE in a 10% gel and then transferred to a polyvinylidene difluoride membrane. After block with 5% BSA, the membrane was incubated with primary antibodies at 4 °C overnight, followed by the corresponding secondary antibodies. The protein bands were visualized by using ECL chemiluminescent detection reagent (GE-Healthcare, Buckinghamshire, UK) and imaged by WSE-6100 LuminoGraph system (ATTO, Tokyo, Japan). The results of optical density ratio of target proteins versus either GAPDH or β-actin are presented as mean ± SEM of at least three different experiments.

### siRNA knockdown

siRNA oligonucleotides against human OR10J5 (ON-TARGET plus smart pool, L-022371-02; Dharmacon, Lafayette, CO, USA) and a pool of nontargeting siRNAs control oligonucleotides (ON-TARET plus Control pool, D-001810-10-05; Dharmacon) were transiently transfected into HepG2 cells using lipofectamin 2000 (Invitrogen), according to the manufacturers’ protocols. The OR10J5 ON-TARGET plus smart pool was a mixture of four different siRNAs. The knockdown efficiency by siRNA was monitored by semiquantitative RT-PCR.

### Statistical analysis

To determine significance of differences between two groups, Student’s t test was used. Statistical analyses were performed in the GraphPad Prism 7 software (GraphPad, San Diego, CA, USA). In all statistical tests, p-values equal to or lower than 0.05 were considered to be statistically significant.

## Electronic supplementary material

Supplementary information

## References

[1] Fleischer, J., Breer, H. & Strotmann, J. Mammalian olfactory receptors. *Front. Cell. Neurosci*. **3** (2009). 10.3389/neuro.03.009.2009 PMC2742912 19753143

[2] Shepard BD, Pluznick JL (2016). How does your kidney smell? Emerging roles for olfactory receptors in renal function. Pediatr. Nephrol..

[3] Kalbe, B. *et al*. Olfactory Receptors Modulate Physiological Processes in Human Airway Smooth Muscle Cells. *Front. Physiol*. **7** (2016). 10.3389/fphys.2016.00339 PMC4972829 27540365

[4] Pluznick JL (2009). Functional expression of the olfactory signaling system in the kidney. P. Natl. Acad. Sci. USA.

[5] Belluscio L, Gold GH, Nemes A, Axel R (1998). Mice deficient in G(olf) are anosmic. Neuron.

[6] Buck L, Axel R (1991). A Novel Multigene Family May Encode Odorant Receptors - a Molecular-Basis for Odor Recognition. Cell.

[7] Pluznick JL (2013). Olfactory receptor responding to gut microbiota-derived signals plays a role in renin secretion and blood pressure regulation. P. Natl. Acad. Sci. USA.

[8] Neuhaus EM (2009). Activation of an Olfactory Receptor Inhibits Proliferation of Prostate Cancer Cells. J. Biol. Chem..

[9] Busse D (2014). A Synthetic Sandalwood Odorant Induces Wound-Healing Processes in Human Keratinocytes via the Olfactory Receptor OR2AT4. J. Invest. Dermatol..

[10] Chang AJ, Ortega FE, Riegler J, Adison DVM, Krasnow MA (2015). Oxygen regulation of breathing through an olfactory receptor activated by lactate. Nature.

[11] Fukuda N, Yomogida K, Okabe M, Touhara K (2004). Functional characterization of a mouse testicular olfactory receptor and its role in cheimosenseng and regulation of sperm motility. Zool. Sci..

[12] Griffin CA, Kafadar KA, Pavlath GK (2009). MOR23 Promotes Muscle Regeneration and Regulates Cell Adhesion and Migration. Dev. Cell.

[13] Kim SH (2015). Expression of human olfactory receptor 10J5 in heart aorta, coronary artery, and endothelial cells and its functional role in angiogenesis. Biochem. Bioph. Res. Co..

[14] de March CA, Ryu S, Sicard G, Moon C, Golebiowski J (2015). Structure-odour relationships reviewed in the postgenomic era. Flavour Frag. J..

[15] Mainland JD, Li YR, Zhou T, Liu WL, Matsunami H (2015). Human olfactory receptor responses to odorants. Sci. Data.

[16] Moon C, Sung YK, Reddy R, Ronnett GV (1999). Odorants induce the phosphorylation of the cAMP response element binding protein in olfactory receptor neurons. Proc. Natl. Acad. Sci. USA.

[17] Kim SY (2015). Phosphoinositide and Erk signaling pathways mediate activity-driven rodent olfactory sensory neuronal survival and stress mitigation. J. Neurochem..

[18] de March CA, Kim SK, Antonczak S, Goddard WA, Golebiowski J (2015). G protein-coupled odorant receptors: From sequence to structure. Protein Sci..

[19] Trott O, Olson AJ (2010). AutoDock Vina: improving the speed and accuracy of docking with a new scoring function, efficient optimization, and multithreading. J. Comput. Chem..

[20] Wong ST (2000). Disruption of the type III adenylyl cyclase gene leads to peripheral and behavioral anosmia in transgenic mice. Neuron.

[21] di Bernardo D (2005). Chemogenomic profiling on a genome-wide scale using reverse-engineered gene networks. Nat. Biotechnol..

[22] Brock A (2014). Silencing HoxA1 by intraductal injection of siRNA lipidoid nanoparticles prevents mammary tumor progression in mice. Sci. Transl. Med..

[23] Ergun A, Lawrence CA, Kohanski MA, Brennan TA, Collins JJ (2007). A network biology approach to prostate cancer. Mol. Syst. Biol..

[24] Xing HM, Gardner TS (2006). The mode-of-action by network identification (MNI) algorithm: a network biology approach for molecular target identification. Nat. Protoc..

[25] Choi, Y., Hur, C. G. & Park, T. Induction of Olfaction and Cancer-Related Genes in Mice Fed a High-Fat Diet as Assessed through the Mode-of-Action by Network Identification Analysis. *Plos One* **8** (2013). 10.1371/journal.pone.0056610 PMC3608641 23555558

[26] Rasineni K, Casey CA (2012). Molecular mechanism of alcoholic fatty liver. Indian J. Pharmacol..

[27] Dodge-Kafka KL (2010). cAMP-stimulated Protein Phosphatase 2A Activity Associated with Muscle A Kinase-anchoring Protein (mAKAP) Signaling Complexes Inhibits the Phosphorylation and Activity of the cAMP-specific Phosphodiesterase PDE4D3. J. Biol. Chem..

[28] Wu HM, Yang YM, Kim SG (2011). Rimonabant, a cannabinoid receptor type 1 inverse agonist, inhibits hepatocyte lipogenesis by activating liver kinase B1 and AMP-activated protein kinase axis downstream of Galpha i/o inhibition. Mol. Pharmacol..

[29] Shaywitz AJ, Greenberg ME (1999). CREB: A stimulus-induced transcription factor activated by a diverse array of extracellular signals. Annu. Rev. Biochem..

[30] Greenberg AS (2011). The role of lipid droplets in metabolic disease in rodents and humans. J. Clin. Invest..

[31] Yap F, Craddock L, Yang J (2011). Mechanism of AMPK Suppression of LXR-dependent Srebp-1c Transcription. Int. J. Biol. Sci..

[32] Zhou GC (2001). Role of AMP-activated protein kinase in mechanism of metformin action. J. Clin. Invest..

[33] Hwahng SH, Ki SH, Bae EJ, Kim HE, Kim SG (2009). Role of Adenosine Monophosphate-Activated Protein Kinase-p70 Ribosomal S6 Kinase-1 Pathway in Repression of Liver X Receptor-Alpha-Dependent Lipogenic Gene Induction and Hepatic Steatosis by a Novel Class of Dithiolethiones. Hepatology.

[34] Herzig S (2003). CREB controls hepatic lipid metabolism through nuclear hormone receptor PPAR-gamma. Nature.

[35] Taylor SS (2008). Signaling through cAMP and cAMP-dependent protein kinase: Diverse strategies for drug design. Biochim. Biophys. Acta.

[36] Liang Y (2016). Hepatic adenylate cyclase 3 is upregulated by Liraglutide and subsequently plays a protective role in insulin resistance and obesity. Nutr. Diabetes.

[37] Li Z (2017). Liraglutide reduces body weight by upregulation of adenylate cyclase 3. Nutr. Diabetes.

[38] Meng JR (2016). Activation of GLP-1 receptor promotes bone marrow stromal cell osteogenic differentiation through beta-catenin. Stem Cell Rep..

[39] Tong T, Shen Y, Lee HW, Yu R, Park T (2016). Adenylyl cyclase 3 haploinsufficiency confers susceptibility to diet-induced obesity and insulin resistance in mice. Sci. Rep..

[40] Peterlin Z, Firestein S, Rogers ME (2014). The state of the art of odorant receptor deorphanization: A report from the orphanage. J. Gen. Physiol..

[41] Young JM, Trask BJ (2002). The sense of smell: genomics of vertebrate odorant receptors. Hum. Mol. Genet..

[42] Baud, O. *et al*. Exchanging ligand-binding specificity between a pair of mouse olfactory receptor paralogs reveals odorant recognition principles. *Sci. Rep*. **5** (2015). 10.1038/srep14948 PMC4598832 26449412

[43] Kajiya K (2001). Molecular bases of odor discrimination: Reconstitution of olfactory receptors that recognize overlapping sets of odorants. J. Neurosci..

[44] Katada S, Hirokawa T, Oka Y, Suwa M, Touhara K (2005). Structural basis for a broad but selective ligand spectrum of a mouse olfactory receptor: Mapping the odorant-binding site. J. Neurosci..

[45] Oka Y, Nakamura A, Watanabe H, Touhara K (2004). An odorant derivative as an antagonist for an olfactory receptor. Chem. Senses.

[46] Oka Y, Omura M, Kataoka H, Touhara K (2004). Olfactory receptor antagonism between odorants. Embo. J..

[47] Folch J, Lees M, Sloane Stanley GH (1957). A simple method for the isolation and purification of total lipides from animal tissues. J. Biol. Chem..

[48] Shepard, B. D., Natarajan, N., Protzko, R. J., Acres, O. W. & Pluznick, J. L. A Cleavable N-Terminal Signal Peptide Promotes Widespread Olfactory Receptor Surface Expression in HEK293T Cells. *Plos One* **8** (2013). 10.1371/journal.pone.0068758 PMC3698168 23840901

[49] Ronnett GV, Hester LD, Snyder SH (1991). Primary Culture of Neonatal Rat Olfactory Neurons. J. Neurosci..

[50] Eswar, N. *et al*. Comparative Protein Structure Modeling Using Modeller. *Current Protocols in Bioinformatics* (John Wiley & Sons, Inc., 2006). 10.1002/0471250953.bi0506s15 PMC4186674 18428767

