# Supplementary material for: Olfactory receptor 10J5 responding to α-cedrene regulates hepatic steatosis via the cAMP–PKA pathway
Source: Sci Rep. 2017 Aug 25;7:9471. doi: 10.1038/s41598-017-10379-x (PMC5573314; doi:10.1038/s41598-017-10379-x)
Supplement: Supplementary file 1 — Supplementary information [file 41598_2017_10379_MOESM1_ESM.pdf]

Supplementary Information

Olfactory receptor 10J5 responding to α-cedrene regulates hepatic steatosis via the cAMP–PKA pathway

Tao Tong[1,*], Sang Eun Ryu[2,*], Yeojin Min[1], Claire A. de March[3,4], Caroline Bushdid[3], Jérôme Golebiowski[2,3], Cheil Moon[2] & Taesun Park[1]

[1]Department of Food and Nutrition, Brain Korea 21 PLUS Project, Yonsei University, 50 Yonsei-ro, Seodaemun-gu, Seoul 120-749, South Korea

[2]Department of Brain and Cognitive Sciences, Daegu Gyeongbuk Institute of Science & Technology (DGIST), Daegu 711-873, South Korea

[3]Institut de Chimie de Nice, Université Nice Sophia Antipolis, Nice cedex 02, France

[4]Department of Molecular Genetics and Microbiology, Duke University Medical Center, Durham, North Carolina 27710, United States

[*]These authors contributed equally to this work.

Correspondence: Taesun Park, Department of Food and Nutrition, Yonsei University, 50 Yonsei-ro, Seodaemun-gu, Seoul 120-749, South Korea. Phone: +82-2-2123-3123; Fax: +82-2-365-3118; E-mail: tspark@yonsei.ac.kr

Supplementary Table

Supplementary Table 1. Primer sequences

Gene description	Sequences (5′→3)	Size(bp)
Olfactory receptor 10J5 *(OR10J5)*	F: CCATCCTTCAAATTGCCTCA<br>R: GATGATGGTGTACGTCACTG	173
Liver X receptor alpha *(LXRα)*	F: CCCTGCTTTGGCAAAGTCTTC<br>R: GATCGAGGTGATGCTTCTGGAG	109
Sterol regulatory element-binding transcription factor 1 *(SREBP1c)*	F: GGTCAGTGTGTCCTCCACCT<br>R: GCAAGGCCATCGACTACATT	188
Adipocyte fatty acid binding protein *(aP2)*	F: TCCTGGCCCAGTATGAAGGA<br>R: TGGCATGGCCAAACCTAACA	108
Fatty acid synthase *(FAS)*	F: TAGTGAGTGGGAAGGTGTAC<br>R: GCAGCTCCTTGTAAACTTCA	126
Stearoyl-CoA desaturase-1 *(SCD1)*	F: TTCGTTGCCACTTTCTTGCG<br>R: CCGGGGGCTAATGTTCTTGT	111
Acetyl-CoA carboxylase 1 *(ACC1)*	F: ACATCCCTACGCTAAACAGA<br>R: CAGCCCATCACTTCATCAAA	187
Peroxisome proliferator-activated receptor gamma *(PPARγ)*	F: AATGACCATGGTTGACACAG<br>R: TTGTAATCTGCAACCACTGG	198
Mitochondrial glycerol-3-phosphate acyltransferase (mtGPAT)	F: CCTTGCCAGACATTTTACCA<br>R: CTCCAAGACAAAGGTTCTGG	158
Carnitine palmitoyltransferase 1 *(CPT1)*	F: CCTTCCCACGGAGAGGTACT<br>R: GGGTACACGCCAGTGATGAT	195
Glyceraldehyde-3-phosphate dehydrogenase (*GAPDH*)	F: CTCTCCAGAACATCATCCCT<br>R: TTGGCAGGTTTTTCTAGACG	157

Supplementary Figures

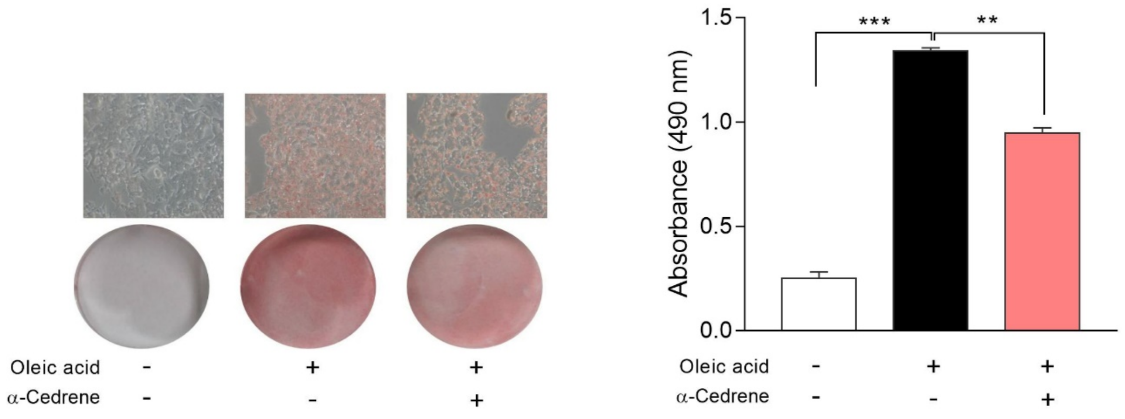

Supplementary Figure 1. α-Cedrene reduces triglyceride concentration in primary hepatocytes. Oil red O staining of primary hepatocytes after stimulation with α-cedrene. Spectrophotometric quantification of Oil Red O-stained primary hepatocytes is presented as means from three independent experiments for each group, and representative photomicrographs (×200) are shown in the left panel. Significant differences between groups are indicated by asterisks; **$P < 0.01$; ***$P < 0.001$.

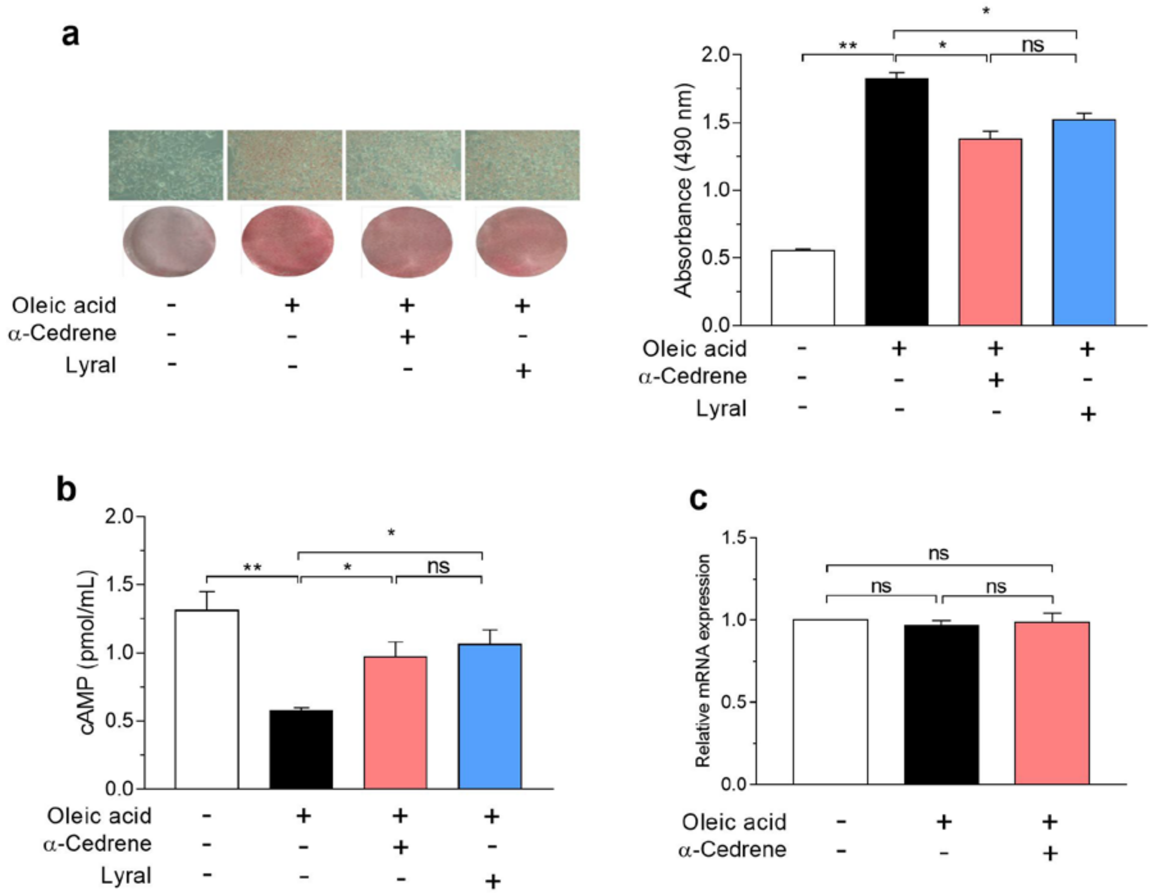

Supplementary Figure 2. In human hepatocytes, lyral induces physiological responses similar to those of α-cedrene. (a) Oil red O staining of HepG2 cells after stimulation with α-cedrene or lyral. Spectrophotometric quantification of Oil Red O-stained HepG2 cell is presented as are means from three independent experiments for each group, and representative photomicrographs (×200) are shown in the right panel. (b) HepG2 cells were incubated with α-cedrene or lyral, and levels of cAMP were determined. (c) mRNA levels of *OR10J5* in HepG2 cells treated with α-cedrene. Significant differences between groups are indicated by asterisks; *$P < 0.05$; **$P < 0.01$.

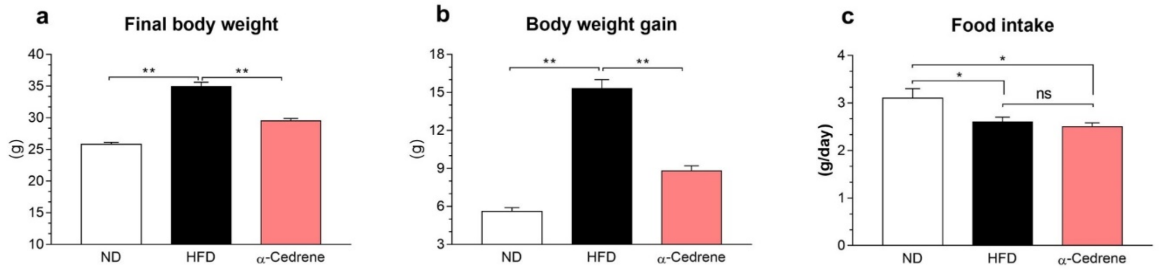

Supplementary Figure 3. α-Cedrene treatment prevents high fat diet-induced body weight gain without affecting food consumption. (a) The final body weights and (b) body weight gain of mice fed the normal diet (ND), high-fat diet (HFD), or 0.2% (w/w) α-cedrene-supplemented diet. (c) Averaged daily food intake of mice. Significant differences between groups are indicated by asterisks; $*P < 0.05$; $**P < 0.01$.

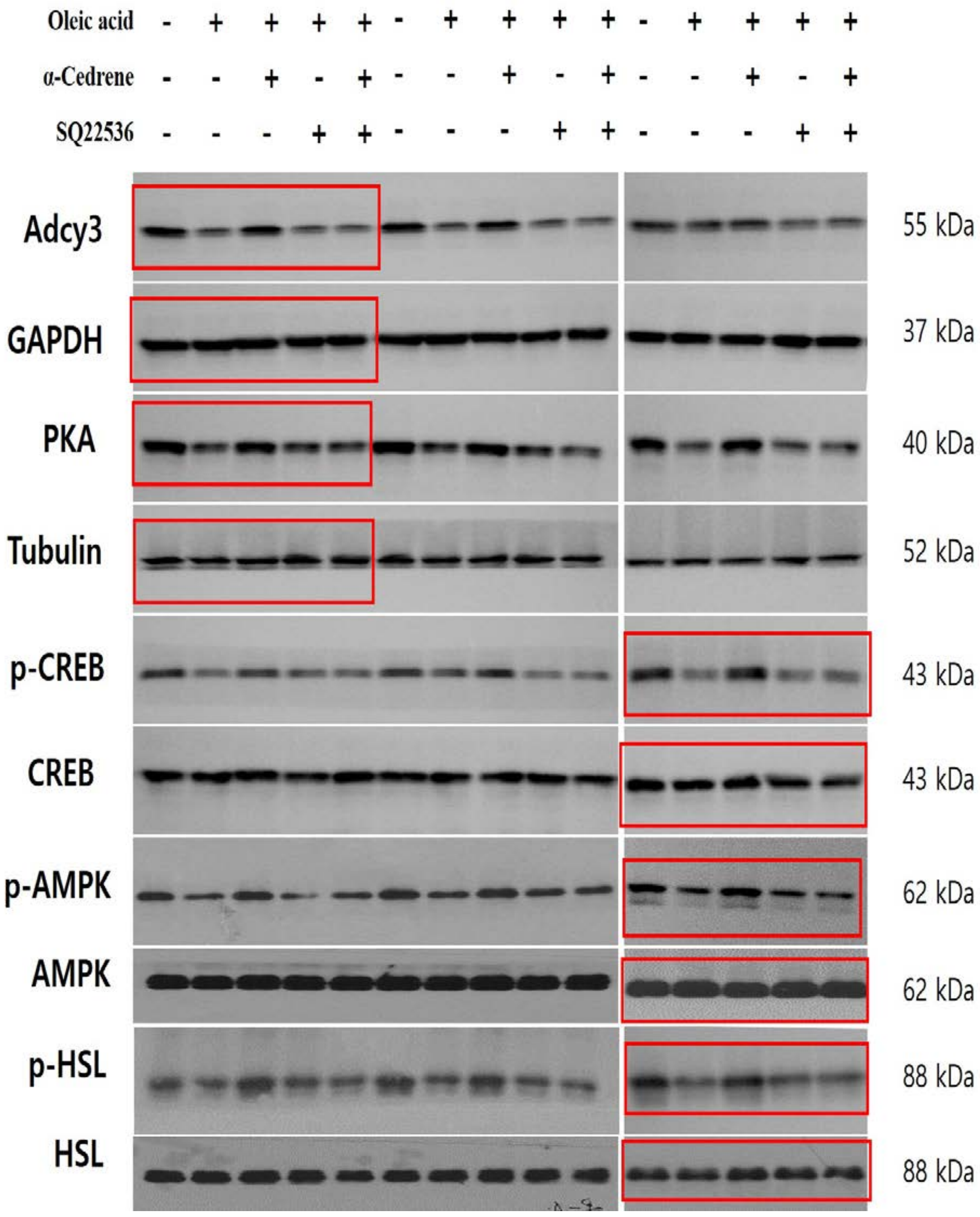

Supplementary Figure 4. Full length western blot membranes relative to Fig 4e. Red boxes indicate the cropping lines used to generate the figures.

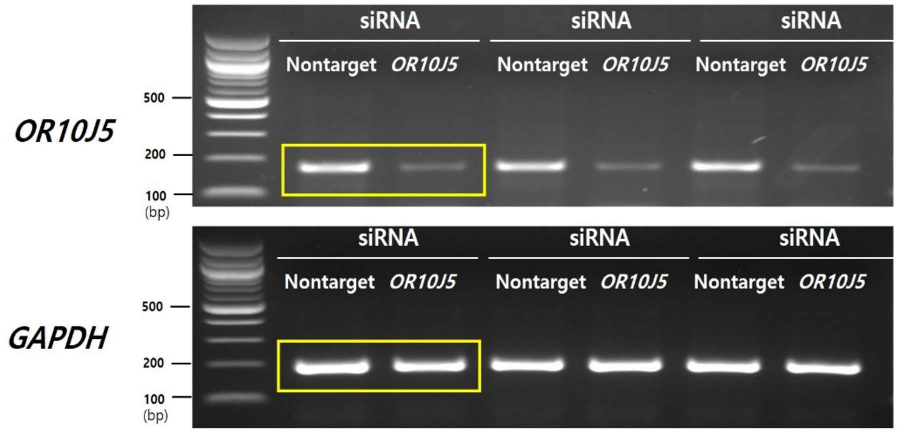

Supplementary Figure 5. Full length agarose gels relative to Fig 5a. Yellow boxes indicate the cropping lines used to generate the figures.

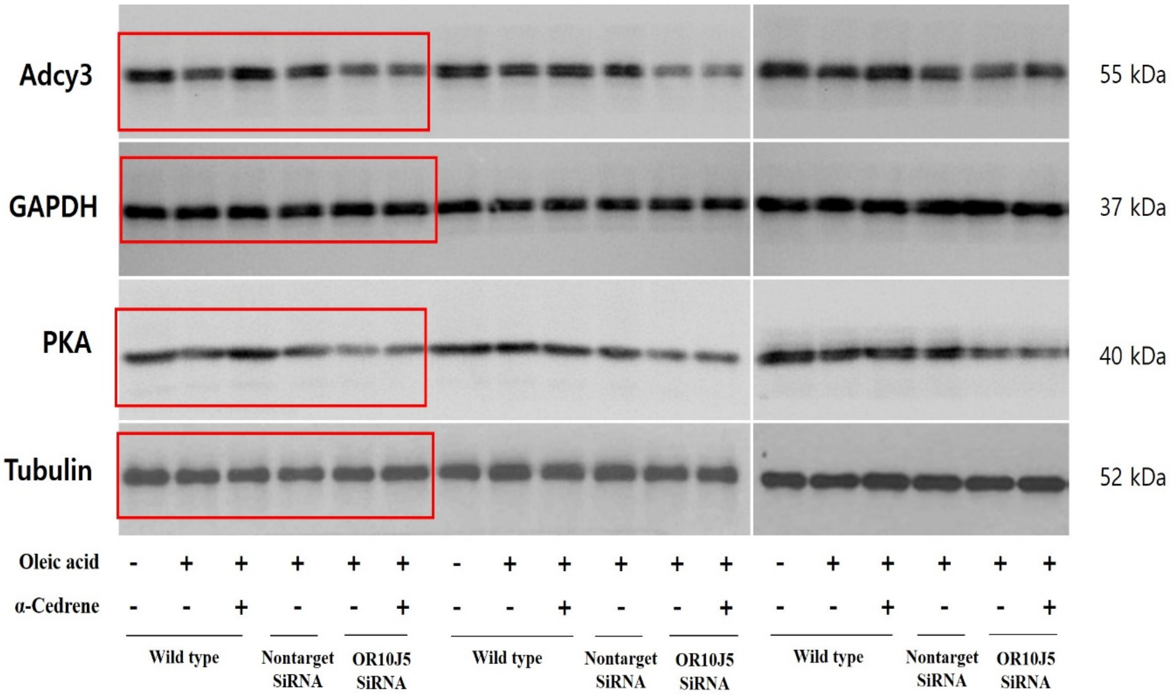

Supplementary Figure 6. Full length western blot membranes relative to Fig 5f. Red boxes indicate the cropping lines used to generate the figures.
